# Association of health checkups with health-related quality of life among public servants: a nationwide survey in Taiwan

**DOI:** 10.1186/s12955-021-01684-1

**Published:** 2021-02-04

**Authors:** Dann-Pyng Shih, Hsien-Wen Kuo, Wen-Miin Liang, Ping-Yi Lin, Pochang Tseng, Jong-Yi Wang

**Affiliations:** 1 Department of Public Health, China Medical University, Taichung, Taiwan; 2 Center for Teaching Excellence, Changhua Christian Hospital, Changhua, Taiwan; 3 Institute of Environmental and Occupational Health Sciences, National Yang Ming University, Taipei, Taiwan; 4 School of Public Health, National Defense Medical Center, Taipei, Taiwan; 5 Department of Health Services Administration, China Medical University, 91 Hsueh Shih Road, Taichung, 40402 Taiwan; 6 Transplant Medicine and Surgery Research Centre, Changhua Christian Hospital, Changhua, Taiwan; 7 Department of Medical Research, China Medical University Hospital, Taichung, Taiwan; 8 Health Promotion Administration, Ministry of Health and Welfare, Taipei, Taiwan

**Keywords:** Preventive health checkup, Preventive health services, Health-related quality of life, SF-36

## Abstract

**Background:**

Preventive health checkups have gained in importance over the last decade. The association of health checkups and the number of diseases with health-related quality of life (HRQoL), including physical and mental health, remains unclear. We sought to investigate the aforementioned association among Taiwanese public servants.

**Methods:**

A cross-sectional survey was conducted using randomized and multistage stratified cluster sampling based on proportional probabilistic sampling. The questionnaires addressed demographics, job characteristics, health behaviors, health status, 3 types of health checkups during the preceding 3 years (government-paid health checkup [GPHC], self-paid health checkup [SPHC], and no health checkup [NOHC]), and physical component summary (PCS) and mental component summary (MCS) scores of the Short-Form Health Survey. In total 11,454 middle-aged public servants were analyzed. A multivariate general linear model (GLM) was used to estimate PCS and MCS scores by using least square means.

**Results:**

Health checkup types were associated with a significant difference in PCS scores among the public servants. Scores of PCS and MCS were both significantly higher in the GPHC group than in the NOHC group for those with no chronic diseases (51.20 vs. 50.66 [*P* = 0.008] and 46.23 vs. 45.58 [*P* = 0.02], respectively). Compared with the NOHC group, both scores of GPHC and SPHC groups were significantly associated with higher PCS scores for public servants with ≥ 2 chronic diseases (46.93 vs. 45.13 [*P* = 0.002] and 46.52 vs. 45.13 [*P* = 0.009], respectively).

**Conclusion:**

In Taiwan, public servants undergoing GPHCs are more likely to report higher PCS scores than are those undergoing SPHCs. It is crucial that encourage periodically using the health checkup to improve health status and HRQoL.

## Background

The advantage of receiving preventive health service is well-documented, suggesting that increasing use of preventive service would benefit population well-being, and would result in savings of personal health care spending [1]. The nationwide preventive service had higher probabilities of early treatment of target chronic diseases and indicated the effectiveness [2]. More new cases of chronic disease were identified among attendees than a matched group of non-attendees [3]. The quality of life of public servants is negatively influenced by chronic health conditions [4, 5] and the out-of-pocket spending grow with the increase in the number of people affected by multiple chronic disease [6].

In Taiwan, public servants not only have preventive adult health checkups covered by the Health Promotion Administration (HPA) but also have government subsidiary health checkups. The package includes basic laboratory tests and general physical examination especially focus on the screening of chronic disease, except for cancer screening or other personalized examinations. Therefore, people have to pay an out-of-pocket payment for any tests not covered by NHI or government [7]. Several studies investigated the characteristics of those who do and do not engage with preventive health checkups [8]. However, little was known about the association between utilization of different kinds of preventive health services and health-related quality of life (HRQoL).

Based on data from Taiwan’s Executive Yuan, the reduction in government manpower is implemented of downsizing and limiting its expansion, the number of national civil manpower has decreased by 5.8% from 2004 to 2015. The estimated proportion of paid public sector employment is 15% of all paid employment in Taiwan [9]. It is much lower than that reported in the OECD countries in 2013 (average rate was about 21%) [10]. These problems may imply that Taiwan's public servants may be insufficient, leading to increased workload. Therefore, it is more important to care about their physical and mental health. However, public servants work for the society and citizens directly, if a government looks after the health of its civil service, the whole of society will benefit. The objective of this study was to compare and discuss HRQoL, including physical and mental health, and its association with different types of health checkups and the number of diseases among public servants in Taiwan.

## Methods

### Study population

This study is the first nationwide survey of public servant workplace health in Taiwan and employs multistage stratified random cluster sampling according to proportional probabilistic sampling (PPS). A two-stage systematic stratified probabilistic sampling combined with a cluster sampling approach were applied to select the study sample. In the first stage, the selected unit is the chief administrative institutions, and each selected unit may have different weights according to the probability proportional to size sampling principle. In the second stage, the selected unit is the subordinate institution, and all staff members of the selected unit were treated as our study subjects. The survey was launched by Taiwan Health Promotion Administration (HPA) to guide the implementation of an intervention program to improve public servant’s health. The results served as a baseline to evaluate the efficacy of the intervention program for public servants. This study obtained ethical approval from the Institutional Review Board of China Medical University Hospital (CMUH105-REC3-091). Participants were informed that their data would be handled confidentially. Information regarding this study was sent to government institutions to encourage public servants to participate in our survey. After agreeing to participate, public servants filled out informed consent forms and completed an online questionnaire. Our study enrolled 21,583 participants, resulting in an overall response rate of 35.8%. Reasons for non-response included unwillingness, vacation, time off, or having insufficient time to fill out the questionnaire, but did not affect the validity of the data. A group of 11,454 public servants aged 40 to 65 years was selected after excluding cases with missing BMI (Body Mass Index) information. Our sample of 11,454 participants had more than 99% power to detect effects size of 0.1 for the significance of the multiple regression analysis with a type I error rate of 0.05. The power was calculated using the statistical package G*Power 3.1.9.2

### Measurement

The online questionnaire included questions related to demographics (gender, age, educational level, monthly income, and marital status), job characteristics (sector type, shift work, and managerial position), health behavior (regular physical exercise and consumption of 5 portions of vegetables or fruit a day), and health status (chronic conditions and obesity). Sectors were categorized as administration, public enterprise, medical services, or public school administration. Job characteristics including shift work and managerial position were crucial considerations. The health behavior section enquired whether respondents exercised more than 3 times a week and consumed 5 portions of vegetable or fruit a day. Obesity was defined using BMI, which is expressed as a weight and height ratio (kg/m^2^). Based on Taiwan HPA guidelines (2012) for Taiwanese adult men and women, BMI was categorized as follows: 18.5 to 24, healthy; 24.1 to 27.0, overweight; and ≥ 27, obese (27–30, mildly obese; 30–35, moderately obese; and ≥ 36, severely obese). Participants were categorized as having a chronic condition if they confirmed ever receiving a diagnosis of one or more of the following diseases: diabetes, hyperlipidemia, hypertension, heart disease, stroke, kidney disease, osteoarthritis, gout, peptic or duodenal ulcer, chronic obstructive pulmonary disease, liver disease, mental disorder, and cancer. Three types of health checkups during the preceding 3 years were classified: GPHC (government-paid health checkup), SPHC (self-paid health checkup), and NOHC (no health checkup). The group with GPHC was defined if they underwent HPA paid health checkups in the previous 3 years or received a subsidy from the government for public servants over the previous 2 years, but they have not received any self-paid health checkups. The participants who underwent any self-paid health checkups were defined as SPHC, and those with no health checkups in the preceding 3 years were defined as NOHC.

### Outcomes

The 36-item questionnaire (SF-36) was used to measure HRQoL for each public servant, which included eight scales: physical functioning, role limitations due to physical problems, bodily pain, general health, vitality, social functioning, role limitations due to emotional problems and general mental health [11, 12]. In Taiwan, this measure has been translated into Chinese, and its validity and reliability have been verified [13]. Scores of physical component summary (PCS), and mental component summary (MCS) were calculated from raw data according to the SF-36 manual. For comparison, the domain scores were calculated to fit a scale of 0–100. The z-scores and factor loadings from the US population were used for the summaries [14, 15]. The average scores of MCS and PCS were used as two primary outcomes of receiving different types of health checkups for cross-sectional HRQoL.

The reliability and validity tests were conducted before the initiation of the survey. The content validity index (CVI) was 81% based on the assessment of content validity by five experts. Two types of interviews also yielded findings that were highly consistent with those of the online questionnaire, which indicates the reliability of the results. In addition, we found consistent results from both high- and low-response groups, and no significant differences were observed in the findings of the two response groups. Our results validated the reliability of the questionnaire with 20,046 participants, which included both a face to face and online questionnaire.

### Statistical analysis

SAS (version 9.4; SAS Institute, Cary, NC, USA) was used to perform the statistical analysis of the data in this study. Descriptive statistics were used to describe the frequency and percentage of study sample variables. A general linear model (GLM) was used to analyze associated factors, mainly health checkup uses and the PCS and MCS scores of the SF-36 with the least square means (LSMs). Several GLMs were used to indicate how different types of health checkup usage affected factors associated with the LSM of the PCS and MCS scores. Analysis of covariance was also used to compare different health checkup–specific LSM scores of the PCS and MCS scores for the three groups across several potential correlates (including demographics, health status, and health behavior factors). The presence or absence of the chronic conditions in public servants influenced their health checkup use and resulted in different LSM of the PCS and MCS scores. A *P* value (two-tailed) of < 0.05 was considered statistically significant. Three regression models were used to adjust sequentially for sets of covariates: Model 1 adjusted for demographic variables, Model 2 adjusted for job characteristic variables, and Model 3 adjusted for health behavior and obesity.

## Results

### Participant characteristics

Our study population consisted of 11,454 participants. Table 1 lists information of all the variables included in this study, with 46.6% male and 53.4% female participants. The independent variables for different types of health checkups were mutually exclusive and categorized into 3 groups depending on health checkup use in the 3 preceding years: GPHC, SPHC, and NOHC, representing 30.1%, 41.4%, and 28.5%, respectively, of the study population. The mean age was 49.4 ± 6.02 years and most participants had a bachelor’s degree. The public servants who didn’t attend any health checkups were statistically more likely to be younger, male, have lower economic status, work in administration sectors, have non-managerial positions, shift-work, unhealthy lifestyles and no history of chronic conditions when compared with those that attended health checkups. This complies with the results of other studies.

**Table 1:** Characteristics of public servants aged 40 to 64 years in Taiwan (N = 11,454)

Variables	Frequency	Utilization rate of Health checkup category			
	n	%	GPHC	SPHC	NOHC
*Health checkup category*					
GPHC	3445	30.1			
SPHC	4747	41.4			
NOHC	3262	28.5			
*Demographics*					
Gender					
Male	5340	46.6	29.2	39.3	31.5
Female	6114	53.4	30.8	43.4	25.8
Age (years)					
40–44	2719	23.7	24.8	34.3	40.9
45–49	3579	31.2	31.0	39.1	29.9
50–54	2732	23.9	30.5	46.9	22.6
55–59	1646	14.4	33.7	46.3	20.0
60–64	778	6.8	35.2	47.7	17.1
Educational level					
Senior high	796	6.9	28.8	41.4	29.8
College	2620	22.9	31.6	38.7	29.7
University	5040	44	30.3	40.4	29.3
Postgraduate	2998	26.2	28.7	45.5	25.8
Monthly Income (NT$)					
60 000 or less	5462	47.7	29.3	37.7	33.0
60 000–80 000	4025	35.1	30.5	42.7	26.8
80 000 and above	1967	17.2	31.5	49.2	19.3
Marital status					
Single	1690	14.7	26.7	32.8	40.5
Married	9181	80.2	30.2	43.2	26.6
Others	583	5.1	37.2	39.0	23.8
*Job characteristics*					
Sector type					
Administration	6120	53.4	28.2	37.8	34
Public enterprise	2635	23	32.2	46.0	21.8
Medical service	896	7.8	38.2	48.2	13.6
Public school	1803	15.8	29.3	43.8	26.9
Shift work					
No	10,203	89.1	30.0	42.1	27.9
Yes	1251	10.9	30.5	36.6	32.9
Managerial position					
No	7948	69.4	29.6	39.40	31.0
Yes	3506	30.6	31.1	46.2	22.7
*Health behavior*					
Regular physical exercise					
No	6418	56	31.2	39.2	29.6
Yes	5036	44	25.0	44.3	30.7
Consumption of vegetables or fruits					
No	7239	63.2	29.6	39.6	30.8
Yes	4215	36.8	30.9	44.7	24.4
*Health status*					
Chronic conditions					
No	7349	64.1	30.5	37.0	32.5
1	2529	22.1	30.2	47.4	22.4
≥ 2	1576	13.8	27.9	52.8	19.3
Obesity					
No	9664	84.4	30.4	41.4	28.2
Yes	1790	15.6	28.6	41.3	30.1

### Associated factors of the PCS and MCS scores

Table 2 lists the different types of health checkups, the mean score of the PCS and MCS scores, and pair comparisons of mean in the crude model and the three other models. In crude Model, scores of PCS in the GPHC group were significantly higher than those who were in SPHC groups while scores of MCS in the GPHC group were significantly higher than those who were in other groups. In Model 1 and Model 2, scores of PCS in the GPHC groups were significantly higher than those who were in other groups while MCS in both GPHC and SPHC groups were significantly higher than those who were in NOHC groups. In Model 3, after adjustments for covariates, GPHC group had significantly higher PCS scores than those in the SPHC group (Mean difference = 0.62, *P* = 0.0001); however, no significant difference was noted for MCS scores.

**Table 2:** Association of 3 types of health checkups with PCS or MCS scores for quality of life among public servants aged 40 to 64 years in Taiwan (N = 11,454) by using a general linear model after adjustment for covariates

Level	CRUDE		Model 1		Model 2		Model 3	
	Mean	*P* value	Mean	*P* value	Mean	*P* value	Mean	*P* value
PCS								
GPHC	51.54		51.46		50.31		50.03	
SPHC	51.02		50.90		49.72		49.41	
NOHC	51.19		50.93		49.86		49.67	
GPHC vs. SPHC	0.52	0.002*	0.56	0.0008*	0.60	0.0003*	0.62	0.0001*
GPHC vs. NOHC	0.35	0.06	0.53	0.004*	0.46	0.01*	0.35	0.05
SPHC vs. NOHC	-0.17	0.32	-0.03	0.84	-0.14	0.42	-0.27	0.12
MCS								
GPHC	44.08		45.14		44.43		45.03	
SPHC	44.18		45.21		44.46		45.00	
NOHC	43.56		44.57		43.93		44.69	
GPHC vs. SPHC	-0.10	0.65	-0.07	0.77	-0.04	0.87	0.03	0.9
GPHC vs. NOHC	0.52	0.04*	0.57	0.02*	0.49	0.05*	0.34	0.16
SPHC vs. NOHC	0.62	0.008*	0.64	0.006*	0.53	0.02*	0.31	0.17

Crude model: only health checkup category in the model

Model 1: Adjusted for gender, age, education, income, and marital status

Model 2: Adjusted for gender, age, education, income, marital status, sector type, shift work, and managerial position

Model 3: Adjusted for gender, age, education, income, marital status, sector type, shift work, managerial position, regular exercise, vegetables or fruit intake, and health status variable including obesity

^*^P < .05

Mean, least square mean; GPHC, government-paid health checkups; SPHC, self-paid health checkups; NOHC, no health checkups; PCS, physical component summary; MCS, mental component summary

### Stratified analysis for chronic conditions

Table 3 shows the difference between PCS and MCS in three groups after stratification for the number of chronic diseases (no chronic disease, having only 1 chronic disease, having 2 or more chronic diseases) using Model 3. In the no chronic disease group, the GPHC group had significantly higher PCS scores than those in the NOHC group (Mean difference = 0.55, *P* = 0.008). Furthermore, those in the GPHC group (Mean difference = 0.65, *P* = 0.02) or SPHC group (Mean difference = 0.81, *P* = 0.003) were significantly higher MCS scores than those in the NOHC group. Only the SPHC group was significantly higher in MCS score than those in the NOHC group (Mean difference = 1.4, *P* = 0.008) for the group with 1 chronic disease. Participants with 2 or more chronic diseases in the GPHC (Mean difference = 1.79, *P* = 0.002) or SPHC groups (Mean difference = 1.39, *P* = 0.009) had significantly higher PCS scores than those in the NOHC group, but no significant difference was noted for MCS score.

**Table 3:** Pair comparison of 3 types of health checkups with PCS and MCS for quality of life in terms of number of chronic diseases among public servants aged 40 to 64 years in Taiwan (N = 11,454) by using a general linear model after adjustment for covariates

Variables	No chronic disease (N = 7349)				Having 1 chronic disease (N = 2529)				Having ≥ 2 chronic diseases (N = 1576)			
	PCS		MCS		PCS		MCS		PCS		MCS	
	Mean	*P* value	Mean	*P* value	Mean	*P* value	Mean	*P* value	Mean	*P* value	Mean	*P* value
GPHC	51.20		46.23		49.81		44.76		46.93		42.06	
SPHC	50.86		46.39		49.66		45.40		46.52		42.22	
NOHC	50.66		45.58		49.73		44.00		45.13		42.19	
GPHC vs. SPHC	0.34	0.08	-0.16	0.56	0.15	0.66	-0.64	0.17	0.41	0.37	-0.16	0.8
GPHC vs. NOHC	0.55	0.008**	0.65	0.02*	0.07	0.86	0.76	0.18	1.79	0.002**	-0.13	0.87
SPHC vs. NOHC	0.21	0.3	0.81	0.003**	-0.07	0.85	1.40	0.008**	1.39	0.009**	0.03	0.97

Mean represents least square mean. *P < .05, ** P < .01

**Fig. 1:**
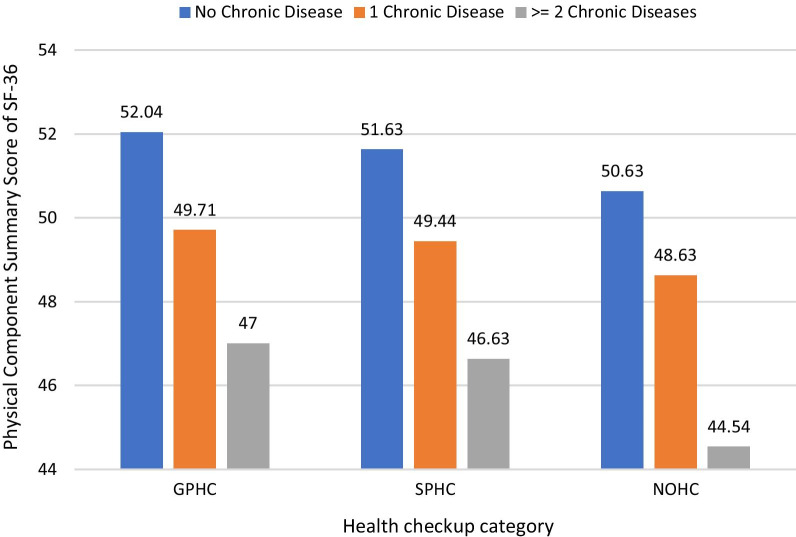
Adjusted means of physical component summary scores for quality of life in different types of health checkups and number of chronic diseases. GPHC, government-paid health checkups; SPHC, self-paid health checkups; NOHC, no health checkups

**Fig. 2:**
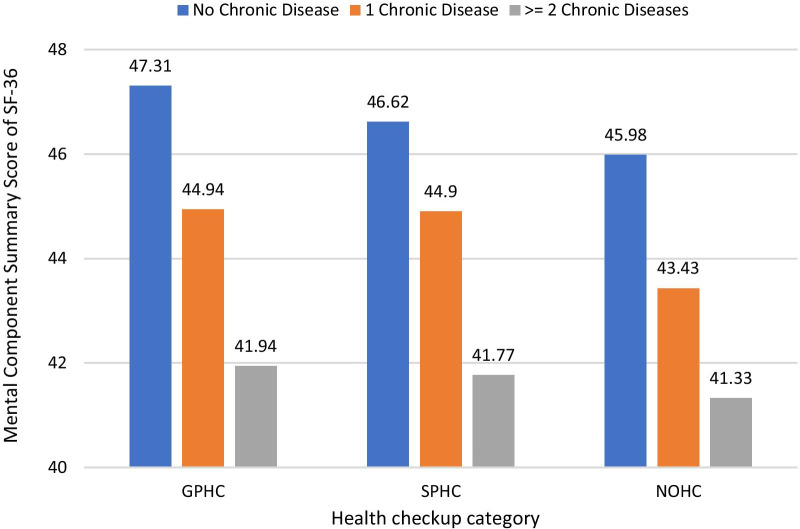
Adjusted means of mental component summary scores for quality of life in different types of health checkups and number of chronic diseases. GPHC, government-paid health checkups; SPHC, self-paid health checkups; NOHC, no health checkups

Figures 1 and 2 illustrate the PCS and MCS scores across different health checkups groups. The trend is the same in both PCS and MCS, where scores show the GPHC group is greater than the SPHC group, and the SPHC group is greater than the NOHC group. This may indicate that public servants undergoing GPHCs are more likely to report higher PCS and MCS scores than are those undergoing SPHCs.

## Discussion

Our findings with respect to health checkup type were associated with a significant difference in PCS and MCS scores among the public servants suggesting that public servants undergoing GPHC had higher PCS scores than are those undergoing SPHC. Furthermore, a declining trend with the increase in number of chronic diseases and scores of HRQoL indicates that as chronic diseases increase, PCS and MCS scores decrease. Similarly, there is a declining trend across GPHC, SPHC and NOHC.

### Factors related to utilization of health checkup

We identified that 71.5% of public servants received health checkups, including GPHC and SPHC, this is greater than the report of 64.5% of citizens aged 40–64 who received health checkups from the Ministry of Health and Welfare [16]. The difference may be accounted for by the fact that only public servants received subsidiaries. In terms of middle or higher socio-economic status, literature has indicated a positive relationship between higher income / social class and utilization rates of preventive services [17, 18].

The findings also showed that non-attendees, NOHC, are younger, male, have a lower educational level, unhealthy lifestyle and multiple chronic conditions compared to attendees; this complies with other study results [19–21]. Related factors like job characteristics, those who work in the administration sector, those in non-managerial positions, and shift work will be less likely to receive health checkups. Since the subsidiary for public servants is in accordance with their job grade, this may result in those with managerial positions attending health checkups and those with shift work failing to schedule checkups with health institutes. The reasons for not attending might include lack of awareness or knowledge, misunderstanding the purpose of the health checkup program, unwillingness to use preventive medicine, time constraints, and difficulties with access to general practices, and doubts regarding clinics as appropriate settings [22, 23].

### Association between health checkup type and HRQoL

With respect to health checkup type were associated with a significant difference in PCS and MCS scores among the public servants suggesting that those undergoing GPHC had higher PCS scores than are those undergoing SPHC. The mean scores of PCS (51.2) and MCS (43.4) in the present study were varied across different countries, US study (48.6, 53.1), and the Whitehall II cohort study (51.2, 51.1), respectively [24]. The score of PCS tended to similar to the Whitehall II cohort study while MCS was lower in our study, perhaps reflecting intrinsic cultural differences compared to the other two studies. Another reason is that public servants have been exposed to a high workload and job stress due to government organizations downsizing, manpower decreasing and the public demand increasing.

Our study shows that the mean scores of PCS and MCS of different types of health checkup decreased from Model 1 to Model 3 adjusted for demographics, job characteristics and life style as well as health status sequentially, and these factors are all related to HRQoL and health checkups. In model 1 and model 2, perhaps items included in GPHC are related to basic physical examination and laboratory tests, the score of PCS in GPHC are higher than the other groups, while the scores of MCS were lower in the NOHC group. This suggests that people with high health consciousness are more likely to have a desire for extensive health check-ups [25]. In addition, self-paid physical checkup programs may complement government-sponsored health screening programs and add value to free or even mild disease screening for health maintenance and help provide good post-checkup care [26–28]. For the SPHC group, the fact that they may already have health problems means they tend to be willing to pay higher out-of-pocket expenses, especially if they have complementary private health insurance [29]. That implies having diseases or good health awareness and perception have an influence to lead people to seek preventive health checkups.

### HRQoL in relation to health checkup and number of chronic diseases

That people with multiple chronic conditions correspondingly had worse HRQoL than those with 1 or no chronic condition and that frequent physical distress was more common than frequent mental distress was consistent with previous studies [5, 30]. We found that public servants with more than 1 chronic disease condition who underwent GPHC are significantly more likely to report higher PCS scores than those undergoing SPHC. The pattern was GPHC was higher than SPHC and NOHC in score of PCS across 3 chronic conditions groups. Different subgroups, including those with no chronic disease, those with one chronic disease, and those with 2 or more chronic diseases, might exhibit different health-seeking behavior and health awareness. On the other hand, both PCS and MCS scores have a significant pair difference for each type of health checkup as the number of chronic conditions increases the score decreases and the two comparisons are statistically significant. Based on health utilization theory, periodic physical health checkup was an enabling factor of awareness of some disease and for disease literacy covering the knowledge of disease screening guidelines and risk factors [31]. A study suggests that low health literacy may affect behaviors necessary for the development of self-management skills [32]. Another consideration is that the specific disease and the diagnosis date were lacking in our data, we may extend our study to investigate the issue. Due to certain chronic diseases having more of an effect on up-to-date screening status than others for different cancers [33]. In some countries, health was an instrumental value exploited as an economic resource not only during periods of well-being but also during illness, by individuals not seeking preventive or timely health care because of the fear of losing their jobs [34].

### Strengths and limitations

This was the first nationwide study of public servants in Taiwan to investigate the association of preventive health checkup profiles with HRQoL, with both mean scores of PCS and MCS. The representative population was selected using PPS and consistent results were validated using both online- and paper-based questionnaires. Our results can aid in the implementation of an intervention program for high-risk groups and consequently be used for follow-up evaluations on the efficacy and effectiveness of the program. However, our study also has several limitations. First, establishing causality was difficult because of the cross-sectional design. Second, complete theoretical factors were not collected for each participant, such as components in the health belief model, self-rated physical factors, and mental outcome in the health utilization model. Third, chronic conditions play a critical role to motivate the seeking of health services as can be noted in Table 3. But the specific diagnosis time and disease need further investigation in future studies. Fourth, we cannot predict if some participants may receive a health checkup program that was covered by an insurance company not an out-of-pocket payment and resulted in the overuse of medical resources. Finally, the differences in preventive health checkup profiles between public servants and the general population encumber the comparison of PCS and MCS scores.

## Conclusion

Public servants who underwent GPHC were more likely to have higher PCS scores than those who underwent SPHC. That implies the present GPHC program does satisfy public servant’s needs, therefore, how to increase the uptake rate is imperative. However, a subgroup of 3 categories according to the number of chronic diseases revealed different results. The association of the number of chronic diseases and preventive health checkups with PCS and MCS scores should be investigated in the future. The usage of government-paid preventive health checkups among public servants in Taiwan merits further emphasis to improve the health outcomes for this population.

## Data Availability

The data underlying the results presented in the study are available upon request from health promotion administration, ministry of health and welfare (Taiwan) after obtaining permission. Website: https://www.mohw.gov.tw/np-125-2.html. The authors did not receive any special access privileges to the data. Interested researchers will be able to access the data in the same manner as the authors.

## Abbreviations

GPHC: Government-paid health checkups;
SPHC: Self-paid health checkups
NOHC: No health checkups
PCS: Physical component summary
MCS: Mental component summary
SF-36: Short-Form 36
HPA: Health Promotion Administration
OECD: Organization for Economic Co-operation and Development
